# Association between GNRI and risk of non-alcoholic fatty liver disease in non-obese individuals: a Chinese longitudinal prospective cohort study

**DOI:** 10.3389/fnut.2025.1574070

**Published:** 2025-05-27

**Authors:** Linlin Shen, Siyu Chen, Haoran Guo, Zhiyan Wang, Jiashu Yang, Ke Chen, Hui Yuan

**Affiliations:** ^1^Department of Clinical Laboratory Center, Beijing Anzhen Hospital, Beijing Institute of Heart, Lung and Blood Vessel Diseases, Capital Medical University, Beijing, China; ^2^Department of Heart Failure Center, Beijing Anzhen Hospital, Beijing Institute of Heart, Lung and Blood Vessel Diseases, Capital Medical University, Beijing, China

**Keywords:** NAFLD, geriatric nutritional risk index, nonobese, nutritional assessment, risk prediction, cohort study

## Abstract

**Background:**

Non-alcoholic fatty liver disease (NAFLD) is a prevalent chronic liver disorder characterized by excessive hepatic fat accumulation without significant alcohol consumption. While obesity is a major risk factor, many non-obese individuals also develop NAFLD. However, research on this subgroup remains limited, and its underlying risk factors are unclear. Nutritional status plays a key role in NAFLD pathogenesis. The geriatric nutritional risk index (GNRI), widely used to assess nutrition, is linked to adverse health outcomes. However, its association with NAFLD in non-obese individuals remains poorly understood.

**Methods:**

A *post-hoc* evaluation was conducted using longitudinal data from the Dryad repository, derived from health screenings of non-obese individuals at Wenzhou People’s Hospital (2010–2014). Participants with baseline NAFLD, alcohol abuse, metabolic disorders, or liver diseases were excluded. The Geriatric Nutritional Risk Index (GNRI) was calculated using serum albumin and weight-to-ideal weight ratios (Lorentz equations). Eligible participants were categorized into quartiles based on GNRI values. NAFLD was diagnosed via ultrasound following Chinese Liver Disease Association criteria. Cox proportional hazards regression (adjusted for confounders in three models), Kaplan–Meier analysis, and restricted cubic splines were employed to evaluate associations between GNRI and NAFLD incidence. Linear trends and subgroup interactions were tested. Statistical analyses were performed using R (v4.0).

**Results:**

The participants in higher GNRI groups had a higher risk of developing NAFLD, even after adjusting for confounding factors. This association was consistent across different body mass index (BMI) levels, although the trend was less significant in the higher BMI group. Furthermore, subgroup analysis revealed a consistent association between GNRI and NAFLD incidence in different sex, age, BMI, and systolic blood pressure (SBP) groups. However, there were some interactions observed in different alanine aminotransferase (ALT) groups.

**Conclusion:**

Our findings suggest that GNRI may serve as a useful indicator for predicting the risk of NAFLD in non-obese individuals, providing valuable insights for early detection and intervention strategies for this condition.

## 1 Background

Non-alcoholic fatty liver disease (NAFLD), which has recently been renamed metabolic-associated fatty liver disease (MAFLD) (1), a range of liver abnormalities, including nonalcoholic fatty liver (NAFL) and nonalcoholic steatohepatitis (NASH). It is estimated to affect approximately 25% of the global population (2). This condition has shown a substantial rise in prevalence, mirroring the global surge in obesity and type 2 diabetes mellitus (3). The 2017 Global Burden of Disease Study revealed that while viral hepatitis continues to be the leading cause of liver-related deaths, NAFLD has emerged as the fastest-growing contributor to liver mortality and morbidity from 2012 to 2017 (4). NAFLD has been associated with an increased risk of developing cardiovascular disease, type 2 diabetes, and chronic kidney disease (5–8).

Although non-alcoholic fatty liver disease (NAFLD) is commonly associated with obesity, it is increasingly being found in non-obese individuals. Based on a comprehensive meta-analysis, the prevalence of NAFLD in non-obese populations has been found to be 40.8%. Notably, in China, this percentage is even higher, reaching 44.3% (9). Therefore, it is imperative to address the urgent issue of evaluating the risk of non-alcoholic fatty liver disease in non-obese individuals.

Geriatric Nutritional Risk Index (GNRI), a predictive marker of malnutrition, was initially designed as a tool to assess the risk of morbidity and mortality in elderly hospitalized patients using simple indicators (10). A lower GNRI value indicates a higher risk of malnutrition. A meta-analysis demonstrated that a lower GNRI is an independent predictor of all-cause mortality and major cardiovascular events in elderly patients with heart failure (11). The GNRI has been identified as the most accurate predictor of readmission for worsening heart failure within one year after discharge in older adults with acute decompensated heart failure in observational research (12). In subsequent applications, it has been found that GNRI can be used to predict the mortality rate of adult patients undergoing hemodialysis across all age groups (13).

GNRI is an assessment tool used to evaluate the nutritional status of older adults. A lower GNRI value indicates a higher risk of negative outcomes. However, in the case of metabolic diseases, such as NAFLD, patients often exhibit higher levels of body mass index. It remains unclear whether higher GNRI levels are linked to adverse outcomes in these patients. Therefore, we aimed to categorize the GNRI levels of non-obese non-alcoholic fatty liver disease patients and examine the influence of different GNRI levels on their prognosis.

## 2 Methods

Data of this study was obtained from the Dryad data repository, which is accessible at http://datadryad.org/. The original study was a longitudinal, observational study, which was designed and conducted by Wenzhou Medical Center of Wenzhou People’s Hospital. Participants in this study where non-obese individuals underwent health screening at the Wenzhou People’s Hospital from January 2010 to December 2014. The original study, which explored the relationship between low density lipoprotein cholesterol (LDL-C) and NAFLD, was previously published (14).

### 2.1 Study design and population

Our study was aimed to explore the association between Geriatric Nutritional Risk Index (GNRI) and incidence of NAFLD in non-obese papulations. GNRI was calculated according to the following formula: GNRI = [14.89 × serum albumin (g/dl) + 41.7 × weight (kg)/ideal body weight (kg)]. The ideal body weight was established using Lorentz’s equations. For men, the ideal body weight is computed as: height-100 - [height-150/4]. For women, it’s calculated as height-100-[height-150/2.5]. If a patient’s actual body weight is greater than the ideal weight, the weight-to-ideal weight ratio is set to 1 (10, 15).

Participants were excluded if they met any of the following criteria: (1) diagnosed with NAFLD at baseline; (2) alcohol abuse (defined as men ≥140 g/week or women ≥70 g/week); (3) any use of antihypertensive, antidiabetic or lipid-lowering drugs; (4) known reason that may cause chronic liver disease; (5) baseline LDL-C > 3.12 mmol/L; (6) BMI over 25 kg/m^2^; (7) those who lost follow-up (14).

### 2.2 Diagnosis of NAFLD

NAFLD was diagnosed by ultrasound by experienced technicians. The criteria of NAFLD were based on the recommendation of the Chinese Liver Disease Association, which contains one mandatory item and four optional items (16). Diffuse enhancement of near-field echoes in the liver area, with gradual attenuation of far-field echoes is necessary. For unnecessary items, including:(1) Unclear visible of the intrahepatic cavity structure; (2) Mild to moderate hepatomegaly with blunt borders; (3) Color Doppler ultrasound shows decreased liver blood flow signals or even difficulty in displaying, but the blood flow distribution is normal; (4) The right hepatic lobe and diaphragm are unclear or incomplete.

### 2.3 Statistical analysis

All eligible participants were divided into four groups by GNRI quartiles. Continuous variables with normal distribution were represented as mean ± SD and analyzed between groups with an analysis of variance. Skewed data were denoted as median (IQR) and assessed using the Kruskal–Walli’s test. Categorical variables, shown as numbers (percentages), were compared between groups via the Chi-square test. Event free rates of patients with newly diagnosed NAFLD were determined using Kaplan–Meier analysis during the follow-up. To assess the relationship between the GNRI groups and the onset of new NAFLD, hazard ratios (HRs) were calculated with Cox proportional hazards regression in three models, providing 95% CIs. The dose–response correlation between GNRI and NAFLD incidence was evaluated using restricted cubic splines. The linear trends between GNRI and NALFD incidence were analyzed using quartiles of GNRI values, with the lowest quartiles of GNRI as the reference. Subgroup analysis assessed the correlation between GNRI groups (treated as a continuous variable) and NAFLD incidence across various subgroups. The *p*-value for interaction was also determined. Within this context, Cox regression determined HRs and *p* values. Statistical significance was set at two-tailed *p* < 0.05. All analyses were performed by software package R (V.4.0; The R Foundation; http://www.R-project.org).

## 3 Results

### 3.1 Participants and baseline characteristics

A total of 14,794 non-obese participants were included in our study. With a median follow-up of 3.2 years, 2035 (13.8%) of these participants developed NAFLD. The baseline characteristics of the participants in different GNRI groups were shown in Table 1. Those in higher GNRI groups had higher body mass index (BMI), higher systolic blood pressure, diastolic blood pressure, ALP, GGT, ALT, AST, ALB, TB, DBIL, BUN, Cr, UA, glucose, TC, TG, LDL, and higher incidence of NAFLD, but lower level of HDL (*p* < 0.001).

**Table 1:** Baseline characteristics of the participants in different GNRI groups.

Variable	GNRI Q1	GNRI Q2	GNRI Q3	GNRI Q4	*p*
*n*	3,674	3,723	3,697	3,700	
Sex, *n* (%)	1982 (53.9)	1944 (52.2)	1833 (49.6)	1979 (53.5)	0.001
age [mean (SD)]	43.94 (15.50)	43.90 (15.29)	43.35 (14.88)	42.75 (14.73)	0.002
BMI [mean (SD)]	19.81 (1.99)	21.19 (1.89)	21.92 (1.69)	22.55 (1.46)	<0.001
SBP [mean (SD)]	117.02 (17.95)	119.64 (16.50)	122.40 (16.52)	124.60 (15.21)	<0.001
DBP [mean (SD)]	69.85 (10.04)	71.68 (9.96)	73.76 (10.15)	75.69 (10.19)	<0.001
ALP [median (IQR)]	68.00 [55.00, 84.00]	67.00 [56.00, 82.00]	70.00 [58.00, 84.00]	73.00 [61.00, 86.00]	<0.001
GGT [median (IQR)]	19.00 [15.00, 27.00]	21.00 [16.00, 29.00]	22.00 [17.00, 32.00]	25.00 [19.00, 37.00]	<0.001
ALT [median (IQR)]	14.00 [11.00, 19.00]	15.00 [12.00, 21.00]	17.00 [13.00, 23.00]	19.00 [14.00, 26.00]	<0.001
AST [median (IQR)]	20.00 [18.00, 25.00]	21.00 [18.00, 25.00]	22.00 [19.00, 25.00]	22.00 [19.00, 26.00]	<0.001
ALB [mean (SD)]	41.72 (2.44)	43.64 (1.74)	44.99 (1.37)	47.24 (1.57)	<0.001
TB [mean (SD)]	11.22 (4.41)	11.81 (4.49)	12.49 (4.96)	13.23 (5.59)	<0.001
DBIL [median (IQR)]	2.00 [1.50, 2.80]	2.10 [1.60, 2.80]	2.20 [1.60, 2.90]	2.20 [1.60, 2.90]	0.005
BUN [median (IQR)]	4.29 [3.50, 5.20]	4.40 [3.70, 5.30]	4.50 [3.80, 5.40]	4.50 [3.83, 5.40]	<0.001
Cr [median (IQR)]	70.00 [62.00, 82.00]	73.00 [63.00, 88.00]	77.00 [65.00, 92.00]	84.00 [71.00, 95.00]	<0.001
UA [mean (SD)]	246.83 (78.79)	269.22 (81.11)	289.89 (82.42)	316.53 (85.07)	<0.001
Glucose [mean (SD)]	5.06 (0.77)	5.12 (0.80)	5.17 (0.78)	5.21 (0.75)	<0.001
TC [mean (SD)]	4.52 (0.77)	4.60 (0.73)	4.68 (0.72)	4.73 (0.73)	<0.001
TG [median (IQR)]	0.92 [0.72, 1.23]	1.03 [0.77, 1.41]	1.14 [0.84, 1.60]	1.30 [0.93, 1.81]	<0.001
HDL [mean (SD)]	1.54 (0.37)	1.47 (0.37)	1.45 (0.36)	1.42 (0.36)	<0.001
LDL [mean (SD)]	2.17 (0.48)	2.25 (0.47)	2.31 (0.45)	2.33 (0.44)	<0.001
GNRI [mean (SD)]	99.08 (2.95)	104.12 (0.99)	107.22 (0.86)	111.29 (2.11)	<0.001
New onset NAFLD, *n* (%)	232 (6.3)	427 (11.5)	558 (15.1)	818 (22.1)	<0.001

### 3.2 Relationship between GNRI groups and incidence of NAFLD

**Figure 1:**
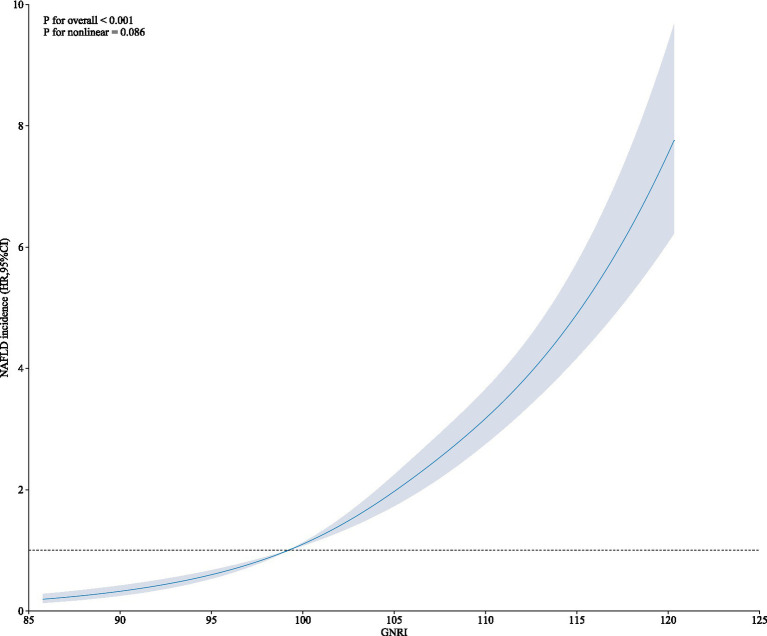
Restricted cubic spline of GNRI and NAFLD incidence.

The HRs and 95%CI of NAFLD incidence in different GNRI groups were presented in Table 2. Participants in higher GNRI groups showed higher risk of NAFLD. When GNRI Q1 group set as reference, the HRs and 95% CI were 1.80 (1.53, 2.11), 2.38 (2.04, 2.77) and 3.58 (3.10, 4.15) in GNRI Q2, Q3 and Q4 groups. After fully adjusting confounders in Model 3 (age, sex, ALP, GGT, ALT, AST, TC, ALB, DBIL, CR, BUN, UA), the association was more significant. The adjusted HRs and 95%CI in GNRI Q2 to Q4 groups were 3.37 (2.62, 4.32), 6.06 (4.59, 8.00) and 11.80 (8.48, 16.43). When GNRI groups set as continuous variables, the adjusted HR and 95%CI was 2.06 (1.88, 2.27), and *P* for trend <0.01. The incidence rates of NAFLD across GNRI quartiles (Q1 to Q4) were 232.00 (6.31%), 427.00 (11.47%), 558.00 (15.09%), and 818.00 (22.11%), respectively. Additionally, we evaluated the association between GNRI and NAFLD incidence across different BMI levels. We found a strong correlation between GNRI and newly diagnosed NAFLD, irrespective of higher or lower BMI, with the association consistently being significant (*p* < 0.05). However, the trend was less significant in the higher BMI group. When GNRI groups set as continuous variables, in the unadjusted model, the HR (95%CI) in higher BMI and lower BMI groups were 1.07 (1.02, 1.12) *P* for trend <0.01 and 1.25 (1.11, 1.40) *P* for trend <0.01, respectively; and in the fully adjusted model, the HR (95%CI) in higher BMI and lower BMI groups were 1.31 (1.13, 1.51) *P* for trend <0.01, and 1.36 (1.06, 1.73) *P* for trend = 0.01. Additionally, as shown in Figure 1, the restricted cubic spline showed a linear effect of GNRI on NAFLD incidence (Figure 1).

**Table 2:** Association between GNRI groups and incidence of NAFLD.

RLP groups	Model 1	Model 2	Model 3
	HR (95%CI) *P* value		
Lower BMI(<=21.45 SD)			
Q1	1	1	1
Q2	1.32 (0.95, 1.83)	1.33 (0.96, 1.84)	1.92 (1.13, 3.27)
Q3	1.53 (1.08, 2.17)	1.57 (1.10, 2.22)	2.19 (1.17, 4.10)
Q4	1.98 (1.35, 2.90)	2.06 (1.40, 3.02)	2.79 (1.25, 6.19)
P for trend	1.25 (1.11, 1.40) *p* < 0.01	1.26 (1.12, 1.42) *p* < 0.01	1.36 (1.06, 1.73) *p* = 0.01
Higher BMI(>21.45)			
Q1	1	1	1
Q2	0.98 (0.81, 1.18)	0.98 (0.81, 1.18)	1.49 (1.10, 2.02)
Q3	0.94 (0.79, 1.13)	0.96 (0.80, 1.14)	1.93 (1.34, 2.79)
Q4	1.16 (0.98, 1.37)	1.17 (0.99, 1.39)	2.42 (1.50, 3.93)
*P* for trend	1.07 (1.02, 1.12) *p* < 0.01	1.08 (1.02, 1.13) *p* < 0.01	1.31 (1.13, 1.51) *p* < 0.01
Total			
Q1	1	1	1
Q2	1.80 (1.53, 2.11)	1.80 (1.53, 2.11)	3.37 (2.62, 4.32)
Q3	2.38 (2.04, 2.77)	2.40 (2.06, 2.80)	6.06 (4.59, 8.00)
Q4	3.58 (3.10, 4.15)	3.63 (3.14, 4.20)	11.80 (8.48, 16.43)
*P* for trend	1.49 (1.43, 1.55) *p* < 0.01	1.50 (1.43, 1.56) *p* < 0.01	2.06 (1.88, 2.27) *p* < 0.01

Model 1 was not adjusted.

Model 2 was adjusted for age, sex.

Model 3 was adjusted for age, sex, ALP, GGT, ALT, AST, TC, ALB, DBIL, CR, BUN, UA.

### 3.3 Kaplan–Meier curve analysis

**Figure 2:**
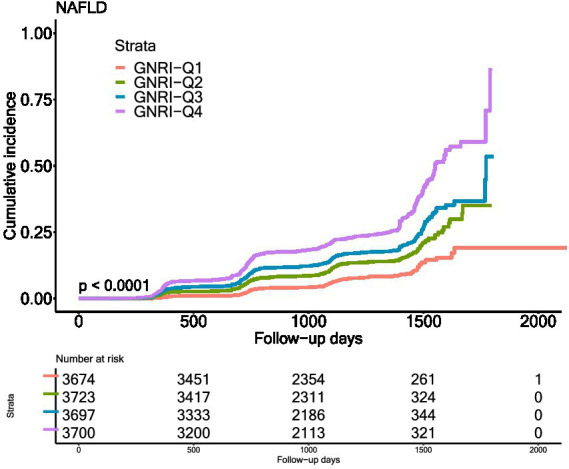
Kaplan–Meier estimation of incidence of GNRI and incidence of NAFLD.

In Figure 2, the Kaplan–Meier curves showed the cumulative incidence of NAFLD in different GNRI groups. The incidence was different in different GNRI groups, with highest in GNRI_Q4 group and lowest in GNRI_Q1 group (*p* < 0.0001).

### 3.4 Subgroup analysis of GNRI and NAFLD risk

Subgroup analysis was conducted in different sex (female vs. male), age (<45 vs. > = 45), BMI (<22 vs. > = 22), SBP (<140, 140–160, and > = 160) and ALT (<16 vs. > = 16) groups. The association between GNRI and NAFLD incidence was consistent in different sex, age, BMI and SBP groups (*P* for interaction >0.05). However, some interactions were observed in different ALT groups. In the lower ALT group, the HR (95%CI) was 2.21 (1.84, 2.67), while in the higher ALT group, the HR (95%CI) was 1.85 (1.65, 2.08) and the *P* for interaction was 0.02 (Table 3).

**Table 3:** Subgroup analysis of the impact of GNRI on NAFLD incidence.

Variables	GNRI groups (per 1 increase)		
	HR, 95%CI	*P* value	*P* for interaction
Sex			0.46
Female	1.91 (1.65, 2.20)	<0.01	
Male	2.20 (1.93, 2.50)	<0.01	
Age			0.99
<45	2.33 (2.01, 2.70)	<0.01	
> = 45	1.86 (1.64, 2.11)	<0.01	
BMI			0.43
<22	1.36 (1.06, 1.73)	0.01	
> = 22	1.31 (1.13, 1.51)	<0.01	
SBP			0.64
<140	2.14 (1.92, 2.38)	<0.01	
140–160	1.81 (1.43, 2.30)	<0.01	
> = 160	1.60 (1.00, 2.55)	<0.01	
ALT			0.02
<16	2.21 (1.84, 2.67)	<0.01	
> = 16	1.85 (1.65, 2.08)	<0.01	

All variables in the model 3 except the studied one was adjusted.

## 4 Discussion

In this observational study, we investigated the association between GNRI and the incidence of NAFLD in non-obese populations. This study is the first to investigate the association between GNRI and NAFLD. GNRI was positively associated with the incidence of NAFLD. After fully adjusting for potential confounders, the risk of newly diagnosed NAFLD increased by 3.6 times for every quarter of increase in GNRI levels. The association remains consistent in the subgroups, except for the differences in ALT levels.

The GNRI is a clinical biological index derived from the NRI, but which is not applicable to the elderly because of difficulties in determining usual weight. Olivier et al. replaced the usual weight in this formula by ideal weight according to the Lorentz formula (WLo), creating a new index called the GNRI.

Previous researches showed that lower GNRI is associated with higher hospitalization rates, longer hospital stays, increased nursing burden, and higher mortality rates. However, there is limited research that specifically examines the predictive value of higher GNRI for metabolic-related diseases.

Participants in higher GNRI groups showed higher risk of NAFLD. After adjusting for confounders, such as age, sex, and various laboratory parameters, the risk of newly diagnosed NAFLD increased by 20% for each tertile of increase in GNRI subgroups. This suggests that nutritional risk, as assessed by GNRI, may be an independent predictor for NAFLD. Furthermore, our study explored the relationship between GNRI and NAFLD incidence across different BMI levels. Interestingly, we found a strong correlation between GNRI and newly diagnosed NAFLD, regardless of higher or lower BMI. This finding suggests that nutritional status may play a crucial role in the development of NAFLD, even in non-obese individuals.

NAFLD has emerged as a substantial global health concern, with a disease burden affecting approximately 25% of the population and an increasing incidence rate. This has attracted significant attention from researchers and healthcare professionals. The pathogenesis of NAFLD is still unclear, involving many factors such as heredity, environment and lifestyle. It is related to obesity, gender, hyperlipidemia, insulin resistance, and diabetes. Thus, it can be considered a metabolic syndrome component. Studies have shown that insulin resistance and lipid metabolism disorder are the central links in the pathogenesis of NAFLD (17).

Our research findings indicate a positive correlation between higher GNRI and an increased incidence of NAFLD. Patients with a higher GNRI are more likely to develop NAFLD within three years, highlighting the importance of lifestyle improvements, weight management, and regular check-ups for early detection and intervention. The seemingly counterintuitive association between elevated GNRI and increased NAFLD risk, because GNRI is conventionally interpreted as a marker of malnutrition, our results are consistent with those of Janicke Visser’s team (18), which identified a paradoxical correlation between higher GNRI and metabolic burden in hospitalized African populations. This paradoxical phenomenon suggests that NAFLD pathogenesis may be linked to overnutrition—a state in which excessive nutritional intake imposes a metabolic burden. These findings underscore the dual role of GNRI as both a nutritional status indicator and a surrogate marker of metabolic dysregulation in non-obese individuals. Given that GNRI relies on weight-dependent calculations, it may inadvertently detect subclinical metabolic dysfunction, such as visceral fat accumulation in individuals classified as non-obese by BMI criteria. Furthermore, in comparison to liver ultrasound, GNRI only requires weight and albumin values, making it easier for patients to monitor their health. This approach enhances awareness and facilitates early intervention to prevent progression to more severe stages. In our study, for non-obese population, higher GNRI means higher risk of NAFLD. The landmark of treatment is still weight loss and improvement of insulin resistance.

Another important finding of our study was that the risk of NAFLD corresponding to GNRI differed significantly according to ALT category. Subgroup analysis revealed consistent associations between GNRI and NAFLD incidence across different sex, age, BMI, and SBP groups. However, we observed some interactions in different ALT groups. The association between GNRI and NAFLD was stronger in the lower ALT group compared to the higher ALT group. This may indicate that nutritional status has a greater impact on individuals with normal or mildly elevated ALT levels. ALT serves as a biomarker of liver injury and is closely associated with NAFLD. However, studies have shown that 25% of NAFLD patients and 19% of NASH patients exhibit normal ALT levels in clinical manifestations, underscoring the need for further validation of ALT’s diagnostic utility in NAFLD/NASH (19). Notably, even among individuals with normal ALT, NAFLD remains significantly linked to hypertension (OR = 2.03, 95% CI: 1.47–2.80; *p* ≤ 0.56) and metabolic syndrome (OR = 1.42, 95% CI: 1.00–2.00; *p* = 0.60). This suggests that metabolic abnormalities (e.g., insulin resistance, lipid peroxidation) may persist in these individuals despite normal ALT. Nutritional factors could directly exacerbate such metabolic dysregulation, promoting hepatic fat accumulation without overt ALT elevation. A cohort study further revealed that NAFLD patients with ALT <0.5 × ULN (upper limit of normal) exhibited the highest all-cause and cardiovascular mortality rates, implying that collapse of metabolic compensatory mechanisms might mask typical liver injury markers (20).

### 4.1 Limitations

A notable limitation of our study pertains to obesity classification. Due to the lack of information on waist circumference, hip circumference, or body composition metrics in the utilized database, our analysis relied solely on BMI ≥ 25 kg/m^2^ as the obesity criterion. This approach may inadequately reflect metabolic risks in the Chinese population, particularly given variations in body composition and fat distribution. Future studies should prioritize the multidimensional integration of anthropometric indicators (e.g., visceral adiposity, waist-to-hip ratio) to improve obesity stratification across diverse populations. Secondly, the present study is limited by its focus on non-obese individuals with NAFLD in China, precluding generalizability to other ethnicities or geographic regions. The applicability of the GNRI may be influenced by ethnic-specific factors. The carbohydrate-dominant dietary patterns (60–70% of energy intake) and prevalent sarcopenic obesity among Chinese populations—characterized by normal BMI with concurrent low muscle mass and elevated adiposity—may bias GNRI’s weight-dependent algorithm toward detecting ectopic fat accumulation rather than true nutritional status (21, 22). This leads to the misclassification of metabolically high-risk individuals as “well-nourished,” a phenomenon less pronounced in populations with higher baseline muscle mass. Furthermore, key covariates such as smoking status, alcohol consumption, and anthropometric measures (waist and hip circumference) were unavailable in our dataset, potentially influencing our findings. Their potential confounding effects are supported by existing evidence. For example, smoking has been shown to significantly exacerbate adverse hepatic outcomes in steatotic liver diseases (23), and structured exercise interventions demonstrate efficacy in ameliorating NAFLD progression (24). These findings underscore the plausible influence of unmeasured lifestyle factors on our results. Given the current scarcity of research exploring the association between GNRI and NAFLD, larger, multicenter studies are warranted to validate the utility and precision of GNRI in predicting NAFLD.

## 5 Conclusion

We investigated the association between GNRI and the incidence of newly diagnosed NAFLD in non-obese populations. After adjusting for conventional risk factors, we found that GNRI level was an independent risk factor for NAFLD. Specifically, a higher GNRI level was associated with a higher risk of developing NAFLD. This suggests that GNRI, a simple and easily accessible parameter, may be useful in identifying the risk of newly diagnosed NAFLD in non-obese individuals. Given the global obesity epidemic, it is crucial to have non-invasive methods for identifying individuals at risk for NAFLD and its complications. GNRI has the potential to be developed into an ideal nutritional assessment tool.

## Data Availability

The datasets presented in this study can be found in online repositories. The names of the repository/repositories and accession number(s) can be found below: the Dryad data repository at: http://datadryad.org/ under the doi: 10.5061/dryad.1n6c4.14.
